# Cell Cycle-Based Molecular Features via Synthetic Lethality and Non-Coding RNA Interactions in Cancer

**DOI:** 10.3390/genes16030310

**Published:** 2025-03-05

**Authors:** Shizheng Xiong, Jiaming Jin, Xinmiao Zhao, Yang Zhao, Zhiheng He, Haochuan Guo, Chengjun Gong, Jiafeng Yu, Li Guo, Tingming Liang

**Affiliations:** 1 State Key Laboratory of Flexible Electronics (LoFE) & Institute of Advanced Materials (IAM), Nanjing University of Posts and Telecommunications, Nanjing 210023, China; 1022173308@njupt.edu.cn (S.X.); 1222014424@njupt.edu.cn (J.J.); 1222014425@njupt.edu.cn (X.Z.); b22100623@njupt.edu.cn (Y.Z.); 1023173006@njupt.edu.cn (Z.H.); 1223014119@njupt.edu.cn (C.G.); 2 Jiangsu Key Laboratory for Molecular and Medical Biotechnology, School of Life Science, Nanjing Normal University, Nanjing 210023, China; 231212007@njnu.edu.cn; 3 Shandong Provincial Key Laboratory of Biophysics, Institute of Biophysics, Dezhou University, Dezhou 253023, China; jfyu1979@126.com

**Keywords:** cell cycle, pan-cancer, molecular subtypes, synthetic lethality, drug targets

## Abstract

Background: The cell cycle, a critical and intricate biological process, comprises various phases, and its dysregulation plays a pivotal role in tumorigenesis and metastasis. The exploration of cell cycle-based molecular subtypes across pan-cancers, along with the application of synthetic lethality concepts, holds promise for advancing cancer therapies. Methods: A pan-cancer analysis was conducted to assess the cell cycle serves as a reliable signature for classifying molecular subtypes and to understand the potential clinical application of genes as potential drug targets based on synthetic lethality. Results: Molecular subtypes derived from cell cycle features in certain cancers, particularly kidney-related malignancies, exhibited distinct immune characteristics. Synthetic lethal interactions within the cell cycle pathway were common, with significant genetic interactions further identifying potential drug targets through the exploitation of genetic relationships with key driver genes. Additionally, miRNAs and lncRNAs may influence the cell cycle through miRNA:mRNA interactions and ceRNA networks, thereby enriching the genetic interaction landscape. Conclusions: These findings suggest that the cell cycle pathway could serve as a promising molecular subtype signature to enhance cancer prognostication and offer potential targets for anticancer drug development through synthetic lethality.

## 1. Introduction

The cell cycle, a ubiquitous and tightly regulated process, is fundamental to various biological functions, including cell growth, proliferation, development, DNA repair, and its involvement in numerous diseases, particularly cancer [1]. Cancer remains a leading cause of death, although advances in diagnostics, clinical management, and novel therapies have gradually improved outcomes. Recent studies have highlighted the clinical value of molecular subtypes based on gene expression and treatment prediction signatures in breast cancer [2,3,4]. Such molecular classifications are also gaining attention in other cancers, including gastric cancer [5], ovarian cancer [6], low-grade glioma [7], and lung cancer [8,9]. Compared to other subtype classifications, cell cycle-based molecular subtypes or risk groups may offer a promising strategy in precision medicine. These subtypes provide critical insights for refining prognostication and treatment predictions in individual patients due to the central role of the cell cycle in tumorigenesis. For example, cell cycle-related molecular classifications have offered novel insights into the prognosis of colon cancer [10], and cell-cycle signature classifiers may expand clinical applications by linking the cell cycle pathway to tumor genomic features [11,12]. Given their potential clinical relevance, cell cycle-based subtypes could significantly enhance both prognostic accuracy and treatment prediction by reflecting their key roles in carcinogenesis.

The cell cycle is tightly regulated by various molecular mechanisms to ensure accurate cell division, including the regulation of cyclin-dependent kinases (CDKs) by cyclins and CDK inhibitors [13]. Additionally, emerging evidence underscores the significant role of non-coding RNAs (ncRNAs) in modulating gene expression related to the cell cycle. Among these, microRNAs (miRNAs), a class of short flexible regulators, can influence cell cycle progression and offer potential clinical applications [14,15,16]. miRNAs affect both the cell cycle and apoptosis by targeting specific mRNAs [17,18,19], thereby contributing to tumorigenesis. These miRNAs interact with numerous target mRNAs, disrupting their expression, and modulate various isomiRs with distinct expression patterns and sequence distributions [20,21,22,23]. Other ncRNAs, particularly long non-coding RNAs (lncRNAs) and circular RNAs (circRNAs), also play critical roles in regulating mRNA expression, especially through acting as miRNA sponges in competitive endogenous RNA (ceRNA) networks. For instance, lncRNA PCAT7 accelerates the cell cycle in non-small cell lung cancer cells through miR-486-5p/CDK4 axis [24], while miRNA and lncRNA networks regulate both the cell cycle and DNA repair [25]. Similarly, lncRNA DLEU2 promotes cell proliferation by perturbing the cell cycle and NOTCH pathway in cervical cancer [26], and STEAP3-AS1 modulates cell cycle progression by influencing *CDKN1C* expression in colon cancer via *STEAP3* [27]. These findings reveal that the regulatory networks governing the cell cycle are more intricate than previously understood, particularly due to the involvement of diverse ncRNAs, which play critical roles in tumorigenesis.

As a ubiquitous and stringently regulated process integral to tumorigenesis, the cell cycle holds immense potential as a pivotal focus for advancements in diagnostic accuracy and anticancer therapies, and the identification of cell cycle-based molecular subtypes and associated clinical implications may offer significant insights for effective anticancer treatment. Herein, a thorough analysis was conducted to identify and characterize cell cycle-based molecular subtypes, examining their expression and functional features. According to the context of synthetic lethality, a concept central to anticancer drug development [28,29,30,31,32,33,34,35], building on the concept of synthetic lethality in anticancer therapy and the potential clinical relevance of the cell cycle pathway in tumorigenesis, further analysis was performed to explore the roles within the framework of synthetic lethality. Furthermore, given the pivotal roles of various ncRNAs in regulation, RNA interaction networks offer a promising approach to unraveling expression profiles of genes involved in synthetic lethal interactions. Our results suggest that cell cycle signature classifiers could serve as valuable subtype indicators in cancers, highlighting the pathway’s central role in tumorigenesis. Understanding synthetic lethal interactions and ncRNA regulators could significantly enhance the clinical application of this pathway.

## 2. Materials and Methods

### 2.1. Data Resources

Relevant genes in the cell cycle pathway were sourced from GSEA [36,37] (http://www.gsea-msigdb.org/gsea/msigdb/cards/KEGG_CELL_CYCLE.html, accessed on 15 October 2023), comprising 125 genes. To explore the molecular characteristics of these genes, sequencing data for miRNAs, mRNAs, mutations, and associated clinical information were retrieved from The Cancer Genome Atlas (TCGA) website using “TCGAbiolinks” [38] (Table S1). To investigate potential genetic interactions with cell cycle genes, experimentally validated synthetic lethal interactions were compiled from the Syn-lethality database [34] and other public references [39,40,41]. Interactions between miRNAs, mRNAs, and miRNAs with lncRNAs were obtained from starBase [42,43], facilitating the exploration of ncRNA roles in gene expression regulation.

### 2.2. Differentially Expressed RNA Profiles and Functional Analysis

Differential expression profiles for various RNAs were analyzed using DESeq2 [44], with significantly deregulated genes identified by |log_2_FC| > 1.2 and padj < 0.05. Functional analysis of these genes was performed to assess their potential contributions to cancer pathophysiology using The Database for Annotation, Visualization, and Integrated Discovery (DAVID) version 6.8 [45] and clusterProfiler 4.0 [46]. Further investigations focused on the role of relevant genes in cancer hallmarks [47], the Cancer Gene Census (CGC) [48,49], core essential genes based on integrated literature data [50,51,52], and oncogenes and tumor suppressor genes (TSGs) from OncoKB [53].

Additionally, Gene Set Variation Analysis (GSVA) scores for the cell cycle gene set were calculated across different cancers using R package GSVA (version 1.44.5) [54] and the GSCA platform [55]. The activity of relevant pathways and drug sensitivity associated with cell cycle genes were also evaluated through the GSCA platform [55].

### 2.3. Identification of Cell Cycle-Associated Molecular Subtypes

Dysregulated genes in the cell cycle pathway were used to assess potential correlations with molecular subtypes across pan-cancers. Unsupervised clustering analysis was performed using the R package ConsensusClusterPlus (version 1.64.0) [56], with 1000 iterations and 80% subsampling (Table S2). Based on the identified subtypes, Kaplan–Meier (K-M) curves were generated to evaluate the overall survival (OS). To further explore the immune characteristics across subtypes, immune scores were calculated using CIBERSORT (version 1.06 ) [57], ESTIMATE (version 1.0.13) [58], and Gene Set Enrichment Analysis (ssGSEA) [54].

### 2.4. ncRNA:mRNA Interactions and Construction of Interaction Network

For synthetic lethal interactions within the cell cycle pathway, relevant miRNAs were selected based on opposite expression patterns and negative correlations with target mRNAs. Following this, associated lncRNAs for the identified miRNAs were screened. An RNA interaction network was constructed using Cytoscape 3.8.2 [59].

### 2.5. Survival Analysis

Survival analysis was conducted to evaluate the prognostic value of relevant genes. Differences between groups were assessed using the log-rank test, and survival outcomes were analyzed through Kaplan–Meier Plotter [60], with integrated results presented.

### 2.6. Statistical Analysis

Statistical differences between groups were assessed using various hypothesis tests, including unpaired *t*-tests, Wilcoxon rank-sum tests, Kruskal–Wallis tests, and trend tests. Spearman correlation analysis was performed to examine expression relationships between miRNAs, mRNAs, and lncRNAs. All statistical analyses were conducted using R (version 4.2.3).

## 3. Results

### 3.1. Expression Profiles of Cell Cycle-Associated Genes

A total of 125 cell cycle-associated genes were identified, with 65 exhibiting significant upregulation across 18 cancer types. The upregulation was more prevalent than downregulation (Figure S1A). Many of these overexpressed genes likely contributed to tumorigenesis. For instance, *CDKN2A* (also known as multiple tumor suppressor I, MTS1) played a role in preventing cell cycle exit, and its protein product may promote colorectal cancer cell metastasis by inducing the epithelial–mesenchymal transition [61]. Some genes, such as *CDKN2B* and *CCNA1*, exhibited inconsistent expression patterns across different cancer types, suggesting dynamic expression across tissues.

**Figure 1:**
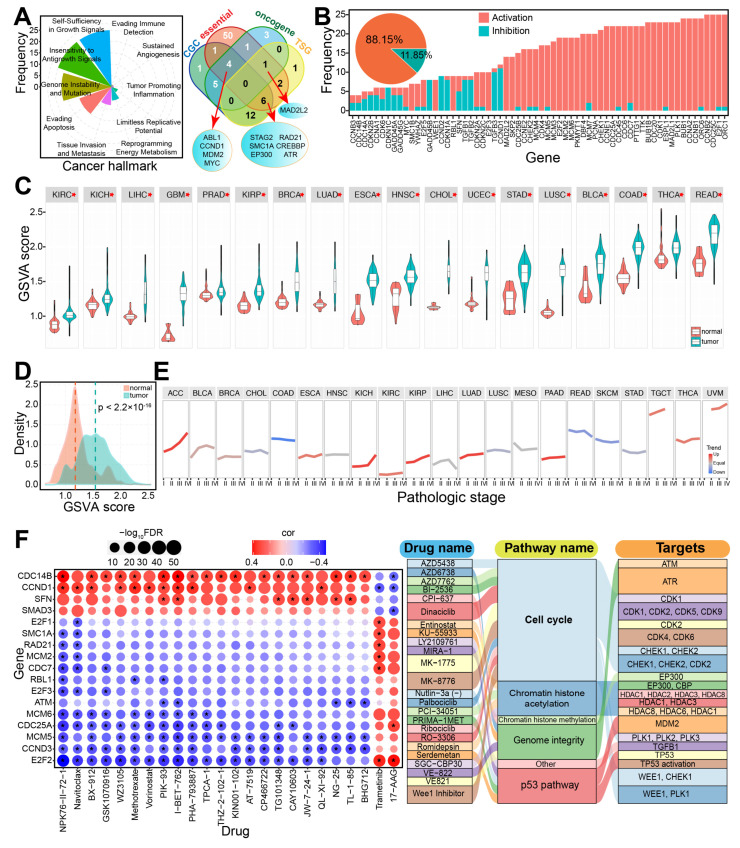
The potential roles of cell cycle-associated genes. (**A**). The cell cycle-related genes play roles in cancer hallmarks and may be identified as CGC, essential, oncogenes, or TSGs. (**B**). Distribution of roles of the 64 screened genes (dysregulated in at least two cancer types), with the overall pie chart distribution presented. (**C**). Distribution of GSVA scores for cell cycle genes across various cancers. * indicates a significant difference between tumor and normal samples (*p* < 0.0001). (**D**). Distribution of GSVA scores for cell cycle genes across all cancer types, comparing all tumor and normal samples. (**E**). GSVA score distribution for different pathological stages in certain cancers. (**F**). Some cell cycle-related genes may serve as potential drug targets. The left panel shows correlations between some cell cycle genes and drugs from the Genomics of Drug Sensitivity in Cancer (GDSC) database. * indicates correlations with values greater than 3.0 or less than −3.0, and FDR < 0.05. The right panel highlights potential relationships between cell cycle-associated genes, involved pathways, and drugs.

Several cell cycle-associated genes were involved in cancer hallmarks (Figure 1A), particularly those related to self-sufficiency in growth signals. Notably, *MYC*, *SMAD3*, and *TGFB1* were implicated in more than four cancer hallmarks. Additionally, some genes were classified as core essential genes, oncogenes, TSGs, or CGC genes, underscoring their roles in the carcinogenic process. Many genes indicated an activation role in the cell cycle pathway, especially in specific cancer types (Figure 1B and Figure S1B).

In addition to promoting apoptosis and regulating the cell cycle pathway, these cell cycle-associated genes were more likely to inhibit other pathways, such as the RAS-MAPK, RTK, PI3K-AKT, and AR hormone pathways (Figure S2A). Further analysis revealed significant differences in gene expression between tumor and normal samples based on GSVA scores across 18 cancer types (Figure 1C,D). These genes were also associated with different pathological stages (Figure 1E) and certain drugs (Figure 1F). The different expression levels in different pathological stages implicated their potential role in classifying molecular subtypes of cancers. Moreover, the observed drug correlations suggested that some of these genes may represent promising drug targets, underscoring their potential clinical relevance in cancer treatment through synthetic lethality-based strategies.

### 3.2. Distinct Molecular Subtypes Associated with Cell Cycle

**Figure 2:**
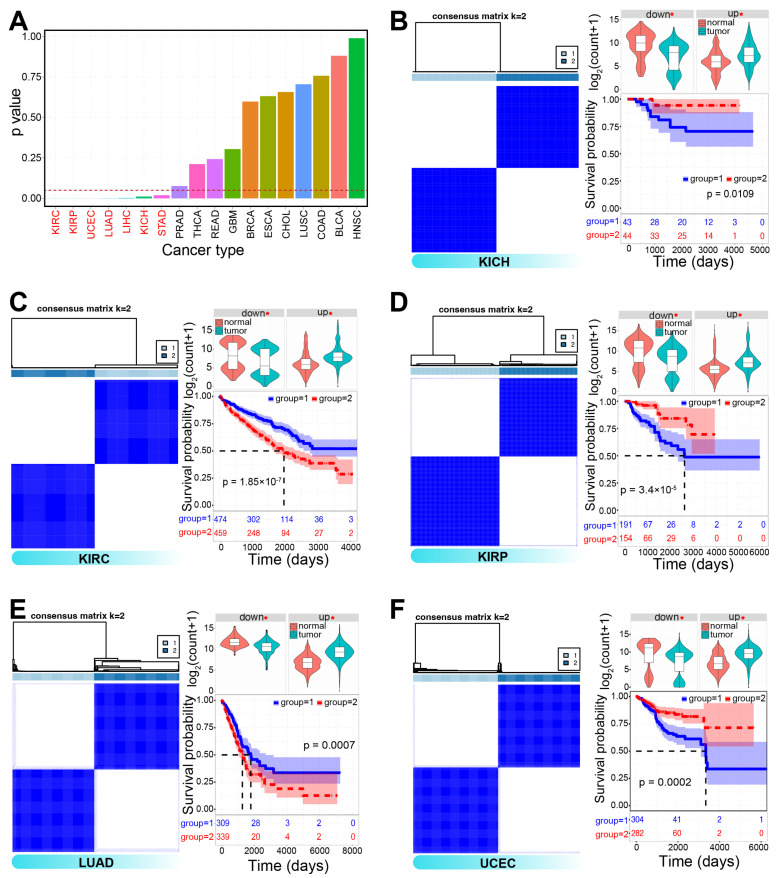
Molecular subtypes derived from cell cycle-associated genes using ConsensusClusterPlus. (**A**). Distribution of *p*-values from clustering analysis of dysregulated cell cycle-associated genes across various cancers. Seven cancer types were divided into two distinct molecular subtypes (*p* < 0.05). (**B**–**F**). Examples of two clusters in specific cancer types based on dysregulated genes. Expression patterns of upregulated and downregulated genes are shown, with a significant survival advantage observed in a specific group. * indicates a significant difference between tumor and normal samples.

**Figure 3:**
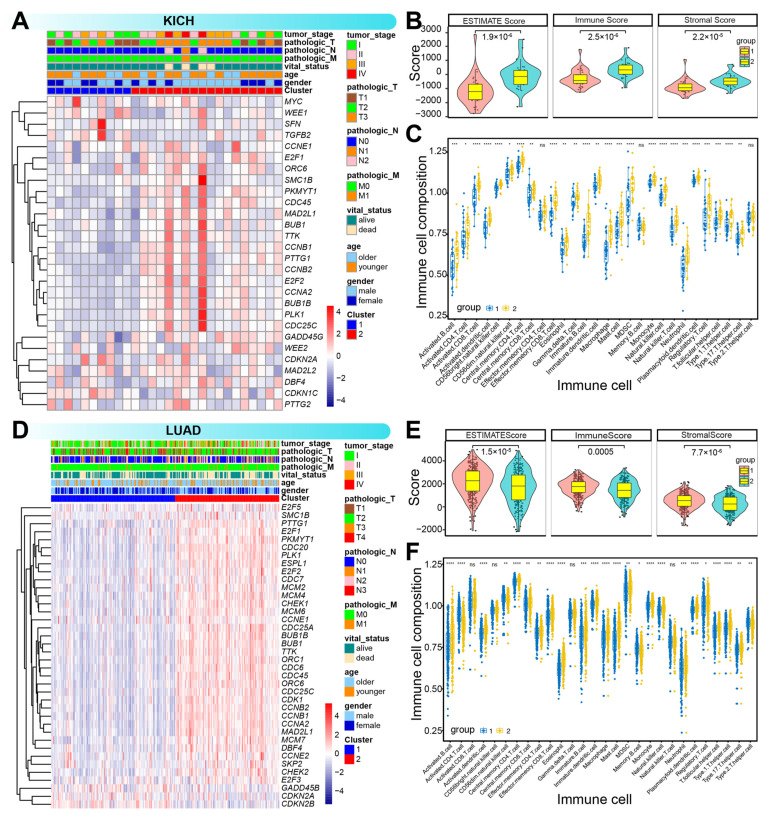
Clinical and immune characteristics of distinct clusters. (**A**–**C**). A heatmap displaying clinical characteristics between the two clusters in KICH (**A**), significant differences in immune-related characteristics (**B**), and comparison of immune cell abundance between the two clusters (**C**). (**D**–**F**). A heatmap displaying clinical characteristics between the two clusters in LUAD (**D**), significant differences in immune-related characteristics (**E**), and comparison of immune cell abundance (**F**) between the two clusters. * indicates *p* < 0.05, ** indicates *p* < 0.01, *** indicates *p* < 0.001, **** indicates *p* < 0.0001, and ns indicates *p* > 0.05.

To explore the potential of cell cycle-related genes as molecular subtype classifiers in tumorigenesis, clustering analysis was performed based on dysregulated genes. Seven cancer types could be categorized into two distinct molecular subtypes, including lung adenocarcinoma (LUAD), uterine corpus endometrial carcinoma (UCEC), three kidney-related cancers—kidney chromophobe (KICH), kidney renal clear cell carcinoma (KIRC), and kidney renal papillary cell carcinoma (KIRP)—, and a specific cluster exhibited a survival advantage over another cluster (Figure 2). Comparative analysis of expression profiles, clinicopathological features, and immune characteristics revealed significant differences between the two clusters (Figure 3 and Figures S2–S4). Notably, in KICH, cluster 2 had higher ESTIMATE scores, as well as elevated immune and stromal scores, and a specific cluster exhibited a survival advantage over another cluster 1 (Figure 3B). Furthermore, distinct immune cell infiltration patterns were observed (Figure 3C), and significant differences in immune checkpoint gene expression were also noted, such as *CD28* and *CD86* in KICH and *CD86*, *HHLA2*, and *PDCD1* in KIRC (Figure S3). These results suggested that cell cycle-associated molecular subtypes may exhibit unique immune and prognostic characteristics, which could aid in prognostic prediction and inform clinical strategies for immunotherapy.

### 3.3. Potential Drug Target Based on Synthetic Lethality

**Figure 4:**
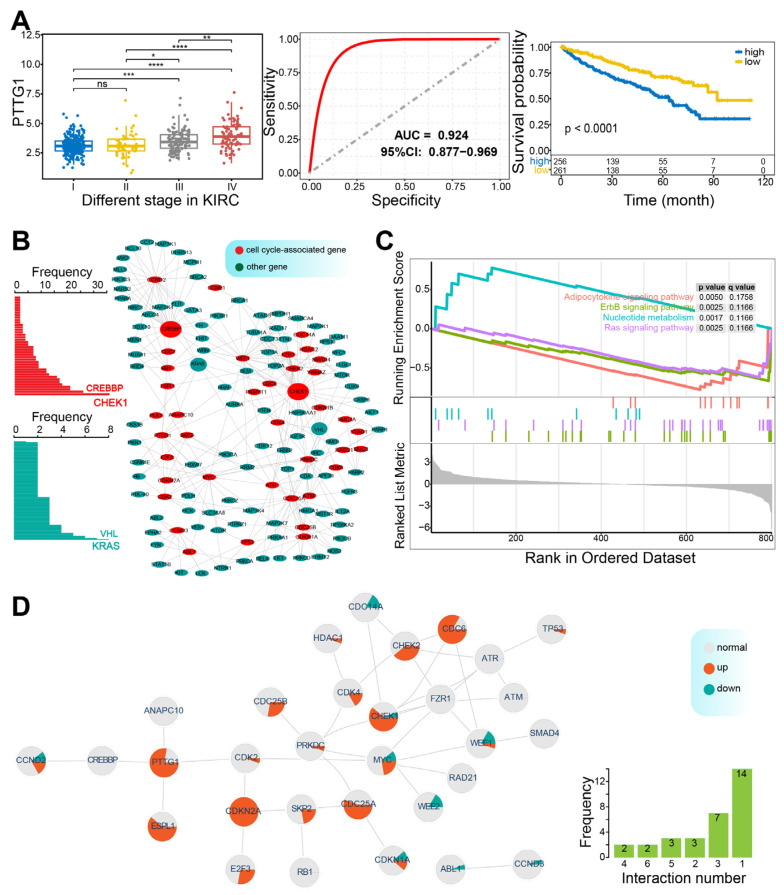
Synthetic lethal interactions associated with cell cycle genes. (**A**). The potential role of PTTG1 in KIRC prognosis. PTTG1, with multiple synthetic lethal interactions (Figure S5), indicates significant expression divergence across different stages of KIRC (left panel) and a strong prognostic ability (middle panel), and its higher expression is associated with a poor prognosis (right panel). * indicates *p* < 0.05, ** indicates *p* < 0.01, *** indicates *p* < 0.001, **** indicates *p* < 0.0001, and ns indicates *p* > 0.05. (**B**). A genetic interaction network for cell cycle-associated genes, where each gene has at least two interactions with others. The frequency of interactions for each gene is also displayed. (**C**). Functional enrichment analysis of genes involved in cell cycle-related synthetic lethality in KIRP via GSEA. (**D**). A network illustrates internal genetic interactions among cell cycle-associated genes, with detailed expression patterns for each gene (node) across different cancers. The number of interactions for each gene is also provided.

A total of 69 genes were identified with synthetic lethal interactions, with *PTTG1* exhibiting the highest number of interactions (470), followed by *MYC* (100) (Figure S5). Star-like patterns were observed for *PTTG1* and *MYC*, highlighting their critical roles and potential for clinical application based on synthetic lethality. The median number of interactions was two, and the mean was 15.13, with notable variability among different genes. *PTTG1*, known to participate in multiple biological processes, plays a pivotal role in tumorigenesis and is associated with clinical outcomes [62]. For instance, *PTTG1* expression showed stage-dependent divergence in KIRC, where it demonstrated a strong prognostic ability (AUC = 0.924), and lower expression levels were linked to better survival (Figure 4A). This gene was also correlated with tumor mutational burden (TMB) and immune-related factors, enhancing its prognostic value for patients with KIRC [63]. Additionally, 776 other genes were involved in synthetic lethal interactions, with *KRAS* exhibiting the most interactions (19), followed by *MUS81* (10). In the genetic network comprising genes with at least two interactions (Figure 4B), which included 40 cell cycle-associated genes and 97 other genes, *CHEK1* showed the highest number of interactions (36), followed by *CREBBP*.

In the context of cell cycle-associated synthetic lethal interactions, several genes were found to play pivotal roles in multiple biological pathways, such as cell cycle regulation, mitotic cell cycle, and the MAPK signaling pathway. Expression pattern analysis in KIRP revealed that these genes were enriched in the Ras signaling pathway, nucleotide metabolism, and ErbB signaling pathway (Figure 4C), all of which were well-established as critical pathways in cancer. These results suggested that cell cycle-related synthetic lethal interactions could have potential clinical applications in anticancer therapies. Notably, some cell cycle genes, including *TP53* and *CREBBP*, exhibited higher mutation frequencies (Figure S6), further supporting the concept of targeting synthetic lethal partners of key driver genes as a viable therapeutic strategy. Furthermore, 38 interactions were identified among cell cycle-associated genes (Figure 4D), involving 31 genes. Some genes showed significant dysregulation in specific cancers, implicating their critical roles in tumorigenesis. The internal synthetic lethal interactions could provide novel drug targets for cancer therapy.

### 3.4. ncRNA-Mediated Regulatory Network Associated with Cell Cycle

**Figure 5:**
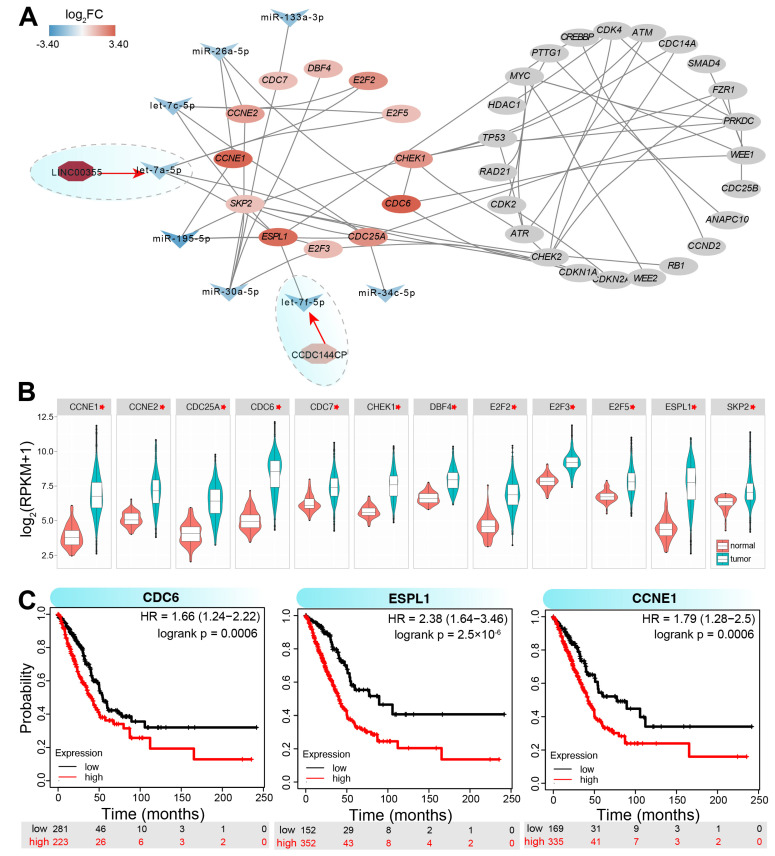
An example of the ncRNA-based genetic interactions in LUAD. (**A**). Some ncRNAs may perturb genetic interactions of cell cycle-associated genes, and the network is constructed using Cytoscape. All miRNA:mRNA and miRNA:lncRNA interactions are verified with opposite expression patterns and negative expression correlations (R < −0.20, *p* < 0.05). (**B**). The detailed expression patterns of the significantly upregulated genes according to Figure 5A. * indicates padj < 0.0001. (**C**). Some upregulated genes have potential prognostic values in LUAD patients.

Given the extensive regulatory roles of ncRNAs, genes involved in internal synthetic lethal interactions within the cell cycle may be negatively regulated by multiple miRNAs (Figure S7). To better understand the ncRNA–mRNA interaction network in LUAD, genes associated with internal synthetic lethal interactions were analyzed for relevant miRNAs. Based on the miRNA–mRNA expression relationships, several miRNAs were identified as key regulators of cell cycle gene expression (Figure 5A), with these miRNAs potentially contributing to the overexpression of target mRNAs. For instance, homologous miRNAs, such as let-7a-5p, let-7c-5p, and let-7f-5p, were found to target certain mRNAs, likely influencing their expression patterns. Subsequently, miRNAs were used to identify related lncRNAs, and two lncRNAs were found to correlate with the RNA interaction network: *CCDC144CP*, which may act as a miRNA sponge for let-7f-5p, and LINC00355, which may sponge let-7a-5p, affecting the corresponding target mRNAs. The mRNA–miRNA–lncRNA network was also detected in other cancer types, suggesting that these RNA interactions form an important regulatory mechanism via the ceRNA network. Dysregulated genes involved in this network exhibited significant upregulation in tumor samples (Figure 5B), indicating that the ncRNA regulatory network played a pivotal role in gene expression regulation. Some of these genes also demonstrated prognostic significance (Figure 5C), with lower expression correlating with better survival outcomes. These results underscore the potential clinical utility of cell cycle-related genes in cancer prognosis and therapy.

## 4. Discussion

The cell cycle, composed of distinct phases, is a complex and tightly regulated biological process, with alterations potentially driving normal cells toward a cancerous phenotype [64]. Aberrant cell cycle activity contributes to tumorigenesis, making this pathway a key target for understanding the fundamental biological mechanisms underlying cancer. Consequently, cell cycle signatures offer promising potential for expanding clinical applications [12]. To investigate the role of the pathway in cancer prognosis, a comprehensive pan-cancer analysis was conducted to explore the roles of cell cycle signatures, assess the potential clinical utility based on the concept of synthetic lethality, and examine the ncRNA regulatory network linked to the cell cycle pathway.

While cell cycle-associated signatures have been reported in some cancers [12,65,66,67], the full scope of signatures involved in the pathway requires further exploration. Herein, leveraging the critical role of the cell cycle in multiple biological processes and tumorigenesis, distinct molecular subtypes were identified, particularly in several kidney-related cancer types. This suggests that cell cycle-associated signatures may serve as effective classifiers for different molecular subtypes. Notably, these subtypes exhibited distinct immune characteristics, indicating that cell cycle-based molecular subtypes may have important clinical implications. Given the central role in tumorigenesis, these cell cycle signatures could provide valuable insights for the development of cancer therapies and the prediction of treatment responses.

Synthetic lethality, a phenomenon involving the simultaneous inactivation or mutation of two non-lethal genes resulting in cell death, has gained significant attention as a novel anticancer treatment strategy, particularly due to its clinical potential in developing targeted therapies aimed at gene pairs of disrupted genes [68,69]. For instance, PARP inhibitors, which are based on the principle of synthetic lethality, have been approved for clinical use, demonstrating the therapeutic value of such interactions. These interactions provide a wealth of new potential targets for cancer treatment. Certain cell cycle genes have been identified with genetic interactions, particularly those involved in multiple interactions. Additionally, internal genetic interactions within the cell cycle have been detected, with some genes showing higher mutation frequencies. Concurrently, ncRNA regulators, including miRNAs and lncRNAs, may contribute to abnormal expression patterns of cell cycle-related genes through RNA interactions, potentially via a ceRNA network. This ceRNA network, widely recognized as a key mechanism for understanding gene regulation, plays a key role in predicting therapeutic responses [70,71]. The ncRNA–mRNA interaction network is essential in regulating the activity of the cell cycle pathway, and ncRNAs themselves hold prognostic value in cancer, suggesting significant clinical implications for these genes. Future studies should focus on the RNA interactions involving diverse RNAs, such as circRNAs and isomiRs derived from miRNA loci, to further expand the associations between the cell cycle pathway and ncRNAs, thus enhancing the clinical application of these pathways in cancer therapy.

Given the molecular features and central roles of the cell cycle pathway in tumorigenesis, dysregulated genes could serve as effective signatures for classifying cell cycle-based molecular subtypes, particularly in kidney-related cancers. The synthetic lethal interactions linked to the cell cycle pathway, especially internal genetic interactions, offer promising drug targets for cancer treatment. Moreover, ncRNAs, through miRNA:mRNA interactions and the ceRNA network, can disrupt the activity of the cell cycle pathway, with short miRNAs and long lncRNAs playing pivotal roles. These findings suggest that the cell cycle pathway may function as a potential subtype signature, providing valuable insights for improving cancer prognostication, treatment prediction, and the development of targeted anticancer therapies.

## Data Availability

The original contributions presented in this study are included in the article/Supplementary Material. Further inquiries can be directed to the corresponding authors.

## Supplementary Materials

The following supporting information can be downloaded at: https://www.mdpi.com/article/10.3390/genes16030310/s1, Figure S1. The overall expression patterns and roles in cell cycle. Figure S2. The potential roles of cell cycle associated genes and clinical characteristics between different groups. Figure S3. The immune characteristics between different clusters in some cancers. Figure S4. The immune characteristics between different clusters in some cancers. Figure S5. The total interaction network in cell cycle-associated genes. Figure S6. The mutation landscape of 69 cell cycle-associated genes in cancers that are detected in synthetic lethality. Figure S7. Primary miRNA-mRNA interaction network based on cell cycle associated genes in Figure 4D. Table S1. Sample sizes in cancer involved in the study based on TCGA. Table S2. The main R package with the parameters used in the study.
