# Supplementary material for: Cell Cycle-Based Molecular Features via Synthetic Lethality and Non-Coding RNA Interactions in Cancer
Source: Genes (Basel). 2025 Mar 5;16(3):310. doi: 10.3390/genes16030310 (PMC11941865; doi:10.3390/genes16030310)
Supplement: Supplementary file 1 [file genes-16-00310-s001.zip › genes-3465681-supplementary.pdf]

Supporting Information

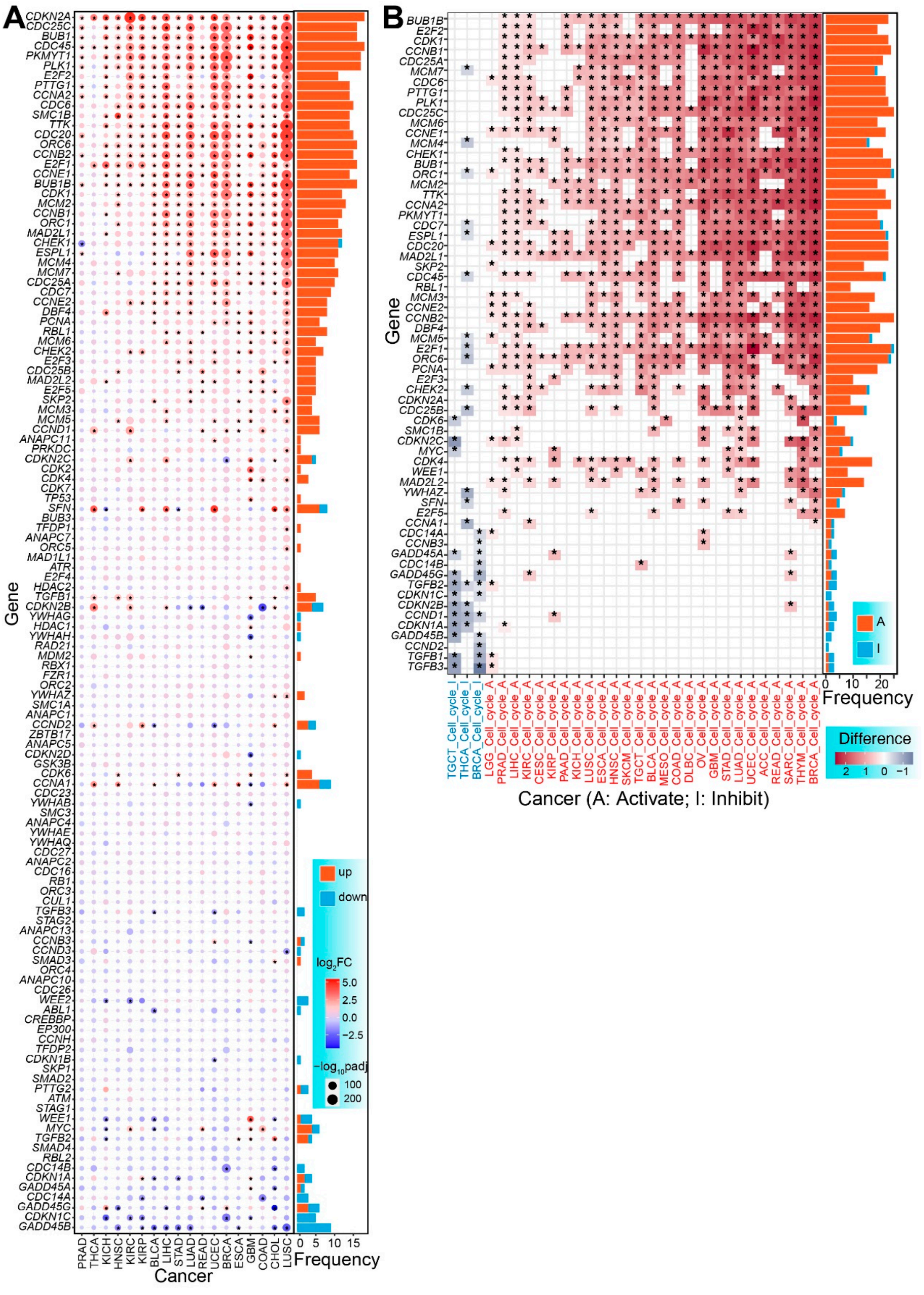

Figure S1. The overall expression patterns and roles in cell cycle. A. The expression patterns of genes associated with cell cycle in cancers. *

indicates significant dysregulated gene (baseMean > 20, $|\log_2 FC| > 1.2$ and padj < 0.05). B. Examples of the detailed activation or inhibition roles of genes in diverse cancers (each of them is involved at least 10 genes).

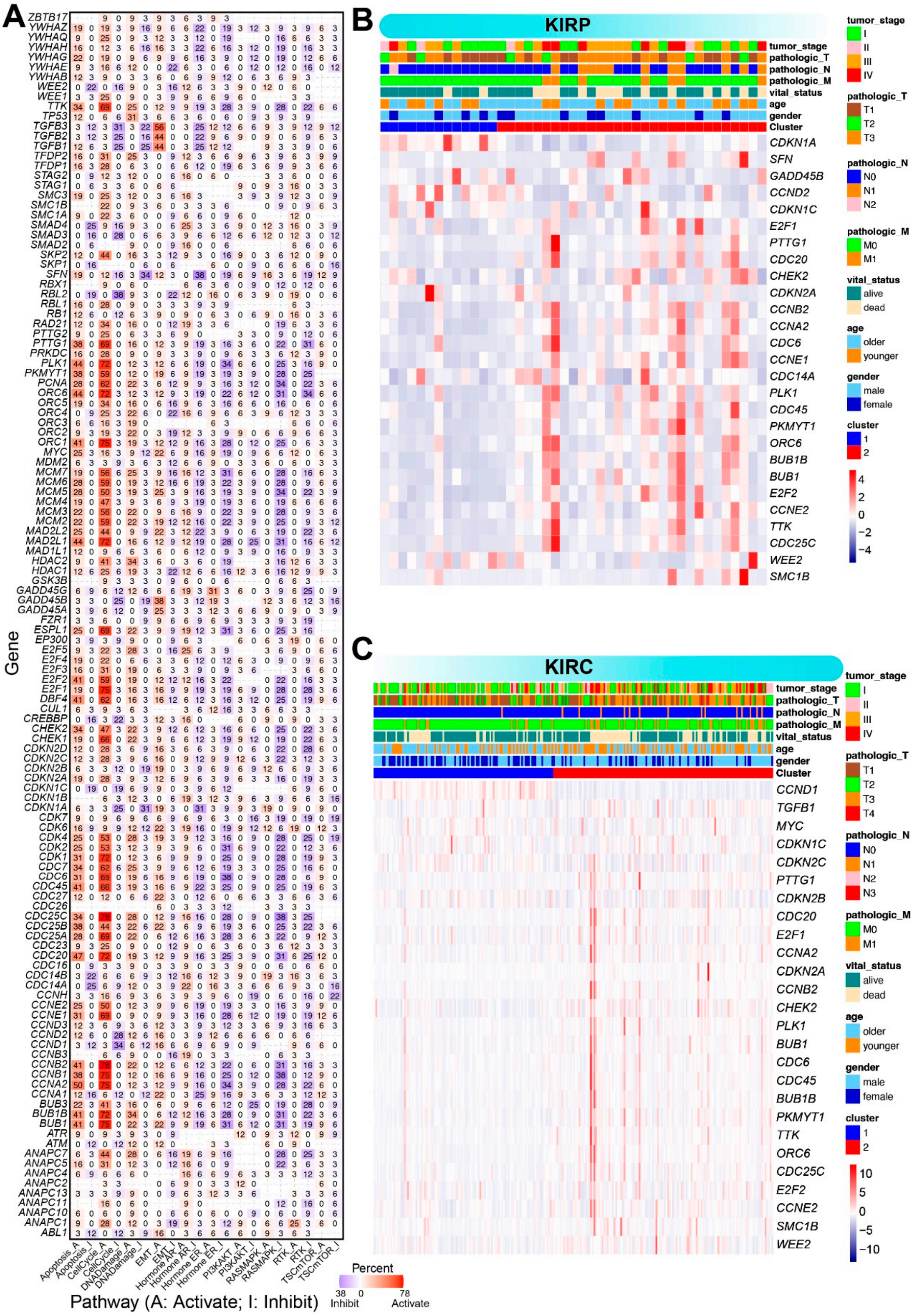

Figure S2. The potential roles of cell cycle associated genes and clinical characteristics between different groups. A. The potential roles of cell cycle-associated genes in different pathways. B. A heatmap shows clinical characteristics between the two groups in KIRP. C. A heatmap shows clinical characteristics between the two groups in KIRC.

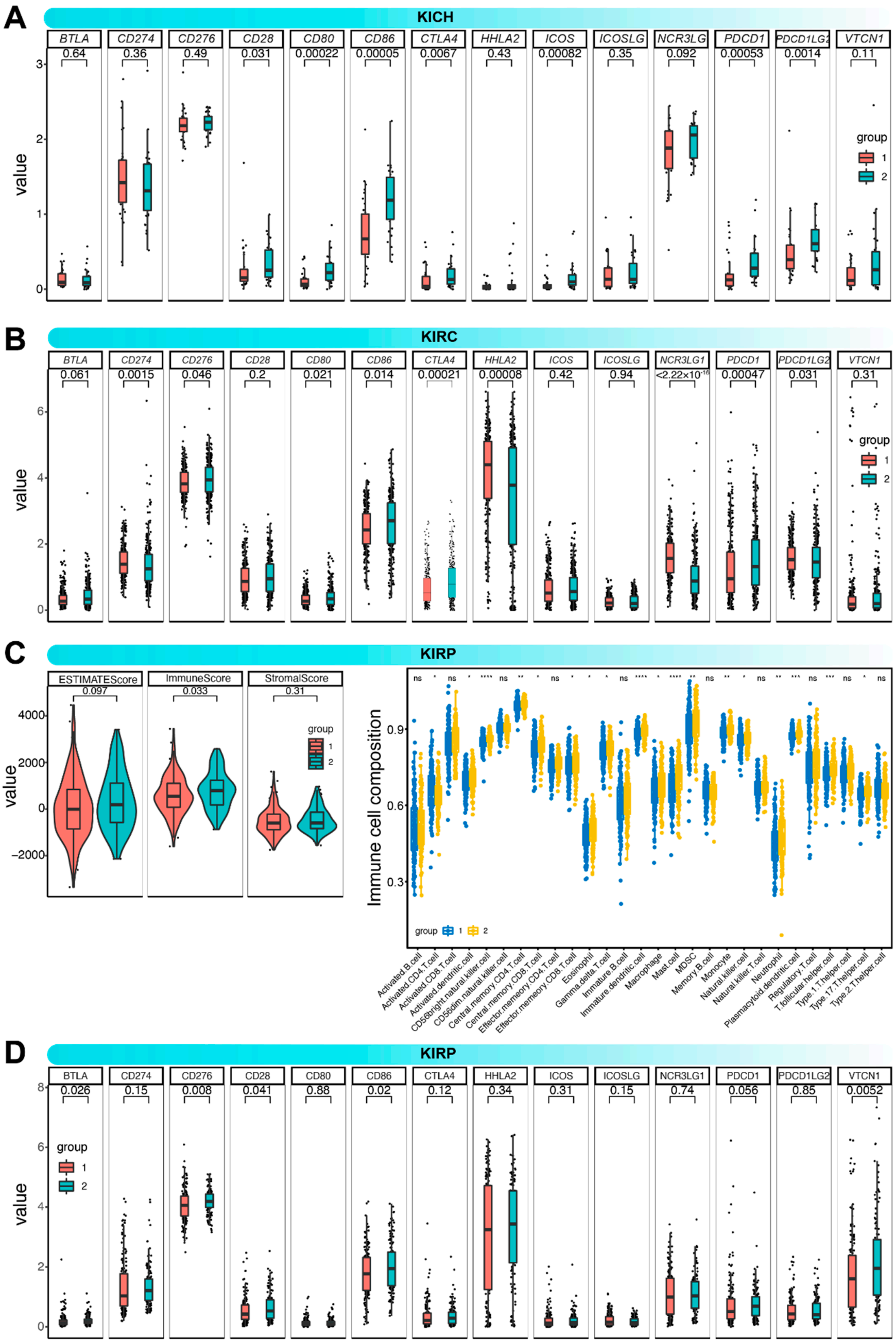

Figure S3. The immune characteristics between different clusters in some cancers. A. The expression pattern of immune checkpoint genes

between the two groups in KICH. The p value for each gene is presented. B. The expression pattern of immune checkpoint genes between the two groups in KIRC. The p value for each gene is presented. C. The two groups in KIRP show significant different immune relevant characteristics. The right picture shows comparisons of the abundances of immune cells in the 2 subtypes. * indicates p < 0.05, ** indicates p < 0.01, *** indicates p < 0.001, **** indicates p < 0.0001, and ns indicates p > 0.05. D. The expression pattern of immune checkpoint genes between the two groups in KIRP. The p value for each gene is presented.

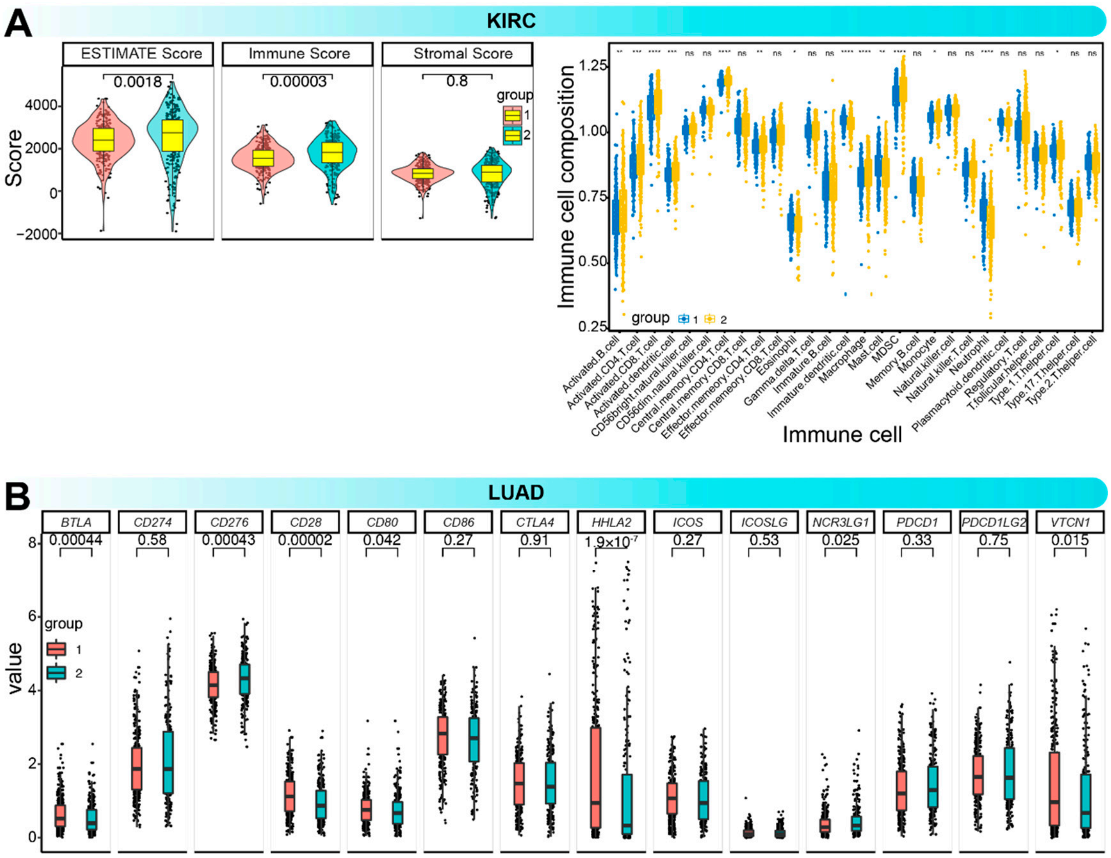

Figure S4. The immune characteristics between different clusters in some cancers. A. The two groups in KIRC show significant different immune relevant characteristics. The right picture shows comparisons of the abundances of immune cells in the 2 subtypes. * indicates p < 0.05, ** indicates p < 0.01, *** indicates p < 0.001, **** indicates p < 0.0001, and ns indicates p > 0.05. B. The expression pattern of immune checkpoint genes between the two groups in LUAD. The p value for each gene is presented.

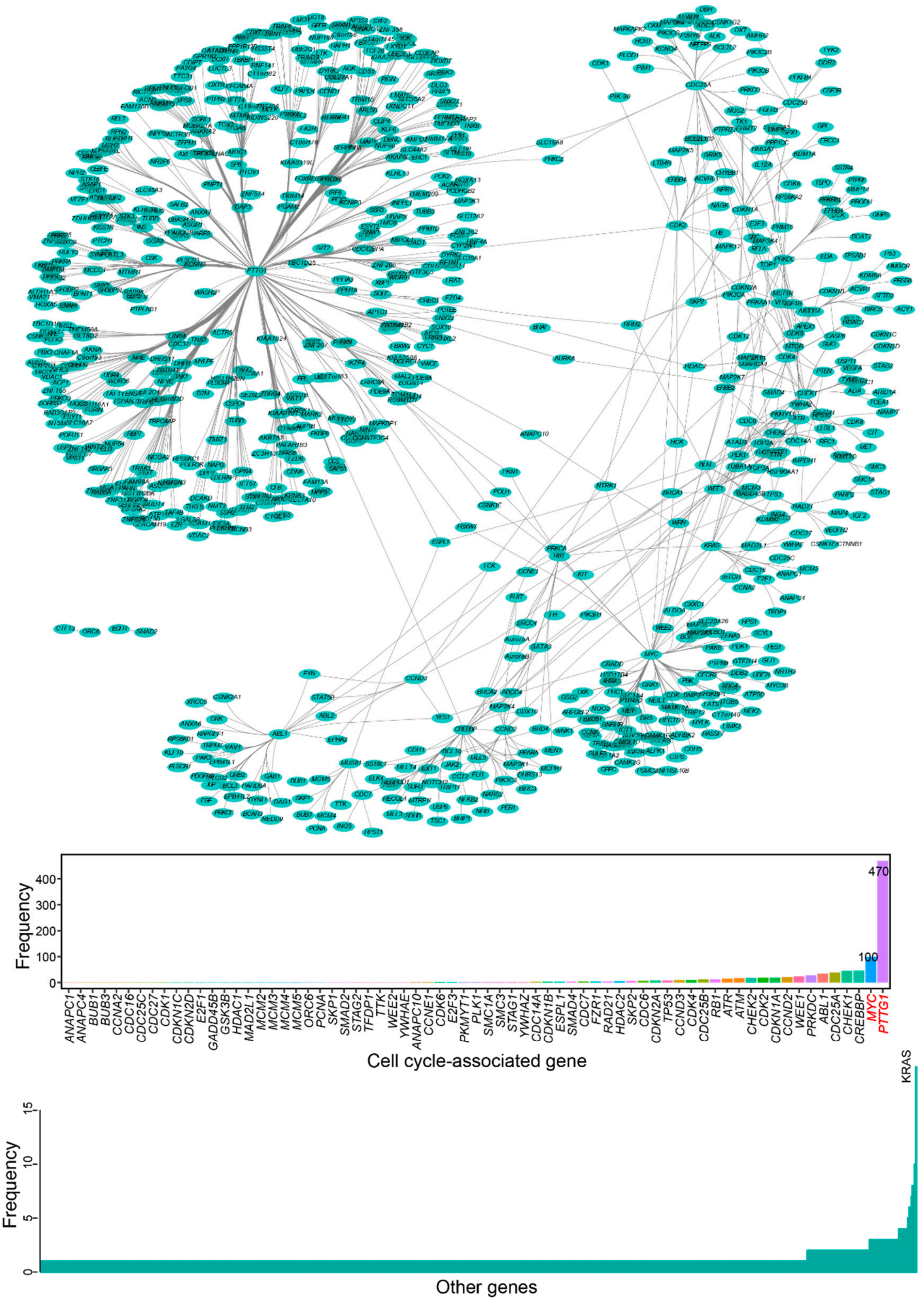

Figure S5. The total interaction network in cell cycle-associated genes. The interaction number frequencies for cell cycle-associated genes and other involved genes are also presented.

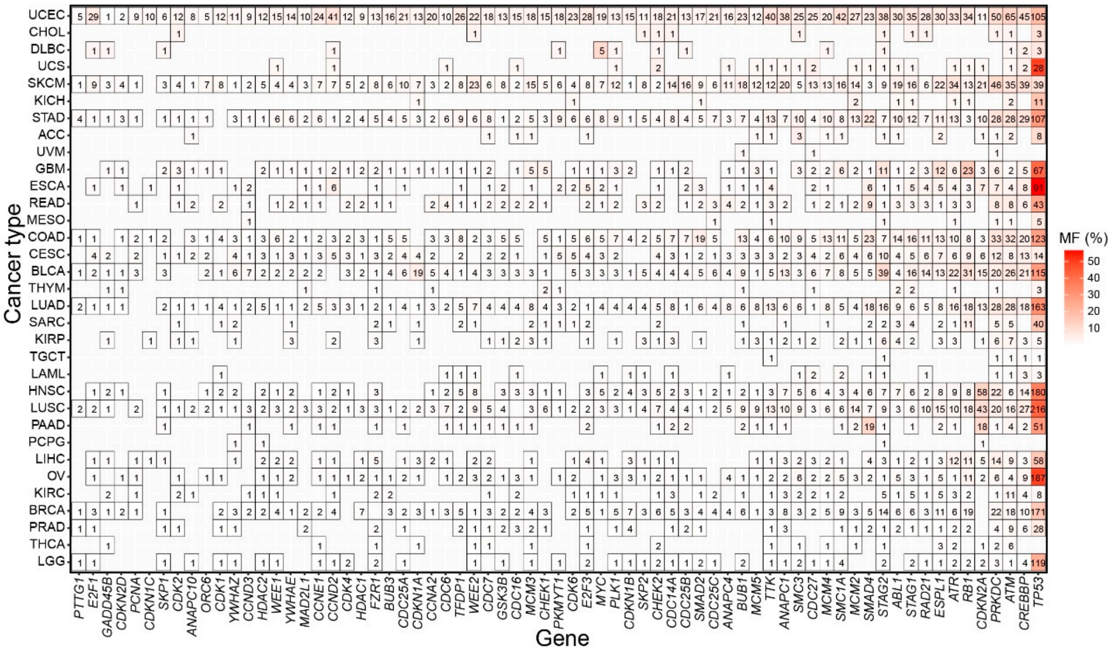

Figure S6. The mutation landscape of 69 cell cycle-associated genes in cancers that are detected in synthetic lethality.

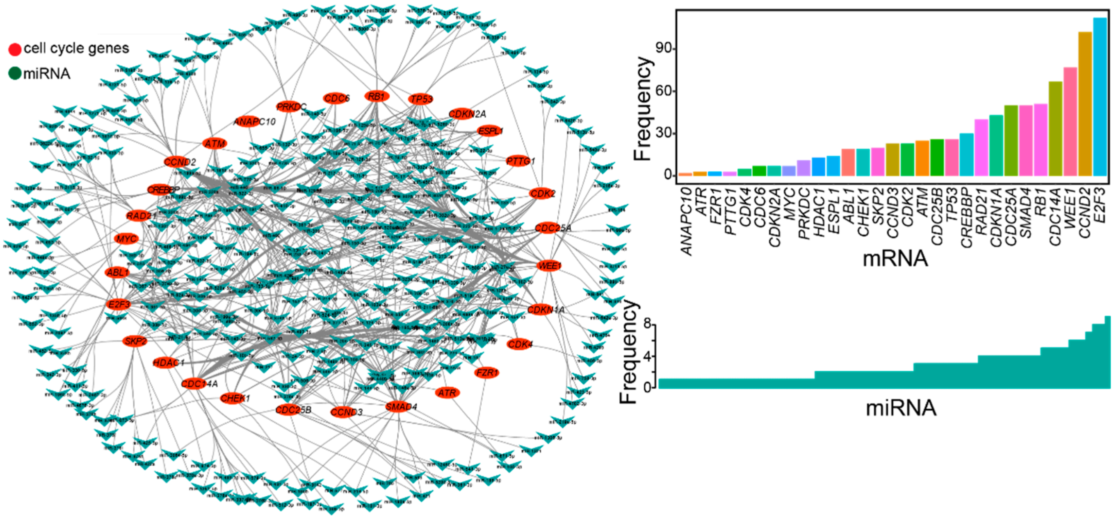

Figure S7. Primary miRNA-mRNA interaction network based on cell cycle associated genes in Figure 4D. All of these miRNA-mRNA interactions are predicted by at least 3 algorithms.

Table S1. Sample sizes in cancer involved in the study based on TCGA.

Cancer type	Full name	Normal	Tumor	Total
ACC	Adrenocortical carcinoma	0	79	79
BLCA	Bladder Urothelial Carcinoma	19	407	426
BRCA	Breast invasive carcinoma	114	1104	1218
CESC	Cervical squamous cell carcinoma and endocervical adeno carcinoma	3	305	308
CHOL	Cholangio carcinoma	9	36	45
COAD	Colon adenocarcinoma	41	288	329

DLBC	Lymphoid Neoplasm Diffuse Large B-cell Lymphoma	0	48	48
ESCA	Esophageal carcinoma	11	185	196
GBM	Glioblastoma multiforme	5	167	172
HNSC	Head and Neck squamous cell carcinoma	44	522	566
KICH	Kidney Chromophobe	25	66	91
KIRC	Kidney renal clear cell carcinoma	72	534	606
KIRP	Kidney renal papillary cell carcinoma	32	291	323
LAML	Acute Myeloid Leukemia	0	173	173
LGG	Brain Lower Grade Glioma	0	530	530
LIHC	Liver hepatocellular carcinoma	50	373	423
LUAD	Lung adenocarcinoma	59	517	576
LUSC	Lung squamous cell carcinoma	51	502	553
MESO	Mesothelioma	0	87	87
OV	Ovarian serous cystadenocarcinoma	0	308	308
PAAD	Pancreatic adenocarcinoma	4	179	183
PCPG	Pheochromocytoma and Paraganglioma	3	184	187
PRAD	Prostate adenocarcinoma	52	498	550
READ	Rectum adenocarcinoma	10	95	105
SARC	Sarcoma	2	263	265
SKCM	Skin Cutaneous Melanoma	1	473	474
STAD	Stomach adenocarcinoma	35	415	450
TGCT	Testicular Germ Cell Tumors	0	156	156
THCA	Thyroid carcinoma	59	513	572
THYM	Thymoma	2	120	122
UCEC	Uterine Corpus Endometrial Carcinoma	24	177	201
UCS	Uterine Carcinosarcoma	0	57	57
UVM	Uveal Melanoma	0	80	80

Table S2 The main R package with the parameters used in the study.

R package	Main parameters
DESeq2	My.dds <- DESeqDataSetFromMatrix(<br>countData = round(countData),<br>colData = myDesign,<br>design = ~ condition)
ConsensusClusterPlus	ConsensusClusterPlus(df, maxK = 20, reps = 1000, pItem = 0.8, pFeature = 1, clusterAlg = "pam", distance="pearson", title=” consensus_cluster”, innerLinkage="complete", plot="pdf")
pheatmap	annotation_col <- data.frame(<br>Cluster=factor(clinical1$group),<br>gender = factor(clinical1$gender),<br>age = factor(clinical1$age),<br>vital_status =factor(clinical1$status),<br>pathologic_M = factor(clinical1$M),<br>pathologic_N = factor(clinical1$N),<br>pathologic_T = factor(clinical1$T),<br>tumor_stage = factor(clinical1$stage))<br>row.names(annotation_col) = colnames(df1)<br>ann_colors = list(Cluster = c("1" = "blue", "2" = "red"),<br>gender=c(MALE="LightSkyBlue",FEMALE="MediumBlue"),

```
age = c(older="LightSkyBlue",younger="DarkOrange"),
vital_status= c(Alive="DarkCyan",Dead="Moccasin"),
pathologic_M = c(M0="green", M1="DarkOrange"),
pathologic_N = c(N0="blue", N1="DarkOrange", N2="pink", N3="red"),
pathologic_T = c(T1="Sienna", T2="green", T3="DarkOrange", T4="red"),
tumor_stage = c("I"="green","II"= "pink","III"= "orange", IV"="red") )
pheatmap(
df,
scale = "row",
cluster_cols = F,
cluster_rows = T,
color = colorRampPalette(c("darkblue", "white", "red"))(200),
show_colnames=F,
annotation_col = annotation_col,
annotation_colors = ann_colors )
```
